# Pan-Cancer Analysis of the Associations of TGFBI Expression With Prognosis and Immune Characteristics

**DOI:** 10.3389/fmolb.2021.745649

**Published:** 2021-10-04

**Authors:** Yun Chen, Han Zhao, Yao Feng, Qin Ye, Jing Hu, Yue Guo, Yunzhi Feng

**Affiliations:** ^1^ Department of Stomatology, The Second Xiangya Hospital, Central South University, Changsha, China; ^2^ Department of Ophthalmology, Eye, Ear, Nose, and Throat Hospital of Fudan University, Shanghai, China; ^3^ Laboratory of Myopia, NHC Key Laboratory of Myopia (Fudan University), Chinese Academy of Medical Sciences, Shanghai, China; ^4^ Shanghai Key Laboratory of Visual Impairment and Restoration, Fudan University, Shanghai, China

**Keywords:** TGFBI, pan-cancer, immune subtype, tumor microenvironment, prognoses, drug responses

## Abstract

Transforming growth factor-beta-induced (TGFBI) protein has important roles in tumor growth, metastasis, and immunity. However, there is currently no pan-cancer evidence regarding TGFBI. In this study, we conducted a pan-cancer analysis of TGFBI mRNA and protein expression and prognoses of various cancer types using public databases. We also investigated the associations of *TGFBI* expression with tumor microenvironment (TME) components, immune cell infiltration, tumor mutational burden (TMB), and microsatellite instability (MSI), along with the *TGFBI* genetic alteration types. The results showed that *TGFBI* expression varied among different cancer types, and it was positively or negatively related to prognosis in various cancers. *TGFBI* expression was also significantly correlated with TME components, TMB, MSI, immune cell infiltration, and immunoinhibitory and immunostimulatory gene subsets. These findings indicate that *TGFBI* participates in various immune responses and it may function as a prognostic marker in various cancers. The findings may be useful for developing immunotherapies that target *TGFBI*.

## Introduction

Transforming growth factor-beta-induced (TGFBI) protein, also known as keratoepithelin or βig-H3, is involved in extracellular matrix (ECM) formation. It can help to bind integrin to ECM proteins such as fibronectin, laminin, and collagen. It was first found in human lung adenocarcinoma cell line. TGFBI protein (68 kDa) has a secretory signal peptide sequence at the N-terminus, a cysteine-rich domain (known as EMI), an Arg-Gly-Asp (RGD) motif, and four internal homologous repeat domains (known as fasciclin 1 [FAS1]) at the C-terminus. The RGD motif and FAS1 domain play key roles in tumorigenesis and development (Skonier et al., 1992). TGFBI is a conserved connective protein that has important roles in cell proliferation, differentiation, adhesion, migration, embryonic development, and inflammation (Kim et al., 2002; Thapa et al., 2007).

In recent years, an increasing number of cancer research has found that TGFBI plays a crucial role in tumor growth, metastasis, and immunosuppression (Wang et al., 2019; Fico and Santamaria-Martínez, 2020; Steitz et al., 2020). The FAS1 domain and RGD motif can bind to integrins *α*3*β*1, *α*5*β*1, and *α*6*β*1 to regulate the PI3K-AKT-mTOR signaling pathway, reduce cell death or promote cell survival, and increase metastasis and angiogenesis (Razumilava and Gores, 2014). TGFBI can also activate the FAK-MAPK-ERK signaling pathway to increase Ca2^+^ and regulate calpain. It thereby stimulates matrix-metalloproteinase (MMP) secretion and alterations in the tumor microenvironment (TME) and cell adhesion, ultimately leading to tumorigenesis, tumor invasion, and metastasis (Ma et al., 2012). Interestingly, TGFBI exhibits dual functions in ovarian cancer (OV) by acting as both a tumor promoter and suppressor. Some studies have shown that upregulated TGFBI can act as a tumor promoter in esophageal squamous cell carcinoma (Ozawa et al., 2016), colon cancer (Ma et al., 2008), gastric cancer (Han et al., 2015), and bladder cancer (Bhagirath et al., 2012). For example, TGFBI is highly expressed in glioblastoma multiforme (GBM) and is related to DC cell infiltration, which is an adverse biomarker of GBM (Yin et al., 2020). In addition, downregulated TGFBI, acting as a tumor suppresser, can cause tumor growth in breast cancer (Li et al., 2012), lung cancer (Wen et al., 2011a), and mesothelioma (Wen et al., 2011b). However, there is still no pan-cancer information about the roles of TGFBI in various types of cancer based on large clinical datasets.

With the rapid development of various public databases, pan-cancer analysis can be used to obtain a profile of any gene of interest, including its associations with cancer (based on analyses comparing tumor and matched normal tissues) and with prognosis, and its potential molecular mechanisms. Here, for the first time, we conducted a pan-cancer analysis of TGFBI mRNA and protein expression and prognoses across various cancer types using several public databases such as, Gene Expression Omnibus (GEO), The Cancer Genome Atlas (TCGA), Oncomine, and Human Protein Atlas (HPA) databases. We also assessed the associations between *TGFBI* expression and immune cell infiltration, immunomodulatory genes, TME components, tumor mutational burden (TMB), tumor microsatellite instability (MSI), and molecular pathways in various types of cancer, along with the types of *TGFBI* genetic alterations.

## Materials and Methods

### TCGA Data and Processing

TCGA database (http://cancergenome.nih.gov) is a landmark public cancer genomics program that, as of 2021, had analyzed molecular characteristics of more than 20,000 primary cancer and normal samples covering 33 cancer types. We used The University of California Santa Cruz (UCSC) Xena website (https://xenabrowser.net/) to collect *TGFBI* data from the TCGA database including RNA-Seq data, clinical data, DNA methylation data, and stemness scores (Goldman et al., 2020). Strawberry Perl (http://strawberryperl.com/; version 5.32.0) was used to obtain the *TGFBI* expression data from the TCGA database and construct a data matrix for further analysis.

### TGFBI Expression Analysis

A comprehensive website TIMER2 (Tumor Immune Estimation Resource, http://timer.cistrome.org/; version 2) was used to systematic analysis the differential gene expression between different cancer types and normal tissues (Li et al., 2017). The “Gene_DE” module was used to compare *TGFBI* expression between various cancer types with adjacent normal tissues using TCGA data.

A comprehensive website UALCAN (http://ualcan.path.uab.edu/analysis.html) can analyze protein expression using data from the Clinical Proteomic Tumor Analysis Consortium (CPTAC) database and the TCGA database (Chandrashekar et al., 2017). The “CPTAC” module of UALCAN website was used to investigate TGFBI protein expression in various tumors and adjacent normal tissues. *p* < 0.05 was considered significant.

An interactive website GEPIA2 (Gene Expression Profiling Interactive Analysis, http://gepia2.cancer-pku.cn/#analysis; version 2) was used to analyze RNA-Seq expression data of cancer tissues and normal samples from the Genotype–Tissue Expression (GTEx) Project and the TCGA database (Tang et al., 2017). The “Expression Analysis-Box Plots” module of GEPIA2 was used to get box plots comparing *TGFBI* expression between cancer and normal tissues. Setting *p* value cutoff <0.01, log_2_ (fold change) cutoff >1, and “Match TCGA normal and GTEx data.” In addition, we used the “Pathological Stage Plot” module to analyze *TGFBI* expression in different pathological stages of various tumors using TCGA data.

A publicly accessible platform Oncomine platform (www.oncomine.org) was used to analyze genome-wide expression, which, as of April 2021, contained 715 datasets and 86,733 samples (Rhodes et al., 2007). We used this platform to compare *TGFBI* expression between various cancer types and adjacent normal tissues using Student’s t-test. We set *p* value cutoff <0.05 and fold change cutoff >2, and selected the top 10% genes.

CCLE (Cancer Cell Line Encyclopedia, portals.broadinstitute.org/ccle/) database provides public access to genomic data, visualization and analysis for over 1,100 cancer cell lines.

### TGFBI Immunohistochemical Analysis

The HPA database (https://www.proteinatlas.org) was used to map all human proteins at the cell, tissue, and organ levels by integrating various omics technologies. We used this database to explore TGFBI mRNA and protein expression data from various cancer types. We also obtained immunohistochemistry images of TGFBI protein expression in cancer tissues.

### *TGFBI* Expression and Cancer Survival

We used the “Survival Map” module in GEPIA2 to gain the overall survival (OS) and disease-free survival (DFS) data correlated with *TGFBI* expression across different cancer types from the TCGA database. The cases were split into high- and low-expression subgroups based on the median expression. The survival data were assessed basing on the Kaplan–Meier (KM) method, with the results being presented as hazard ratios, 95% confidence intervals, and *p* values of log-rank tests.

The PrognoScan database (http://dna00.bio.kyutech.ac.jp/PrognoScan/index.html) and Cox regression analysis (with *p* < 0.05 indicating significance) were used to verify the relationships between *TGFBI* expression and various survival outcomes in a pan-cancer analysis, including OS, DFS, relapse-free survival (RFS), disease-specific survival (DSS), distant metastasis-free survival (DMFS) and distant recurrence-free survival (DRFS) (Mizuno et al., 2009).

The “survival” and “survminer” R package were used to analyze the correlation of the *TGFBI* expression and OS, DSS, disease-free interval (DFI), and progression-free interval (PFI) across all TCGA tumors. We computed log-rank *p*-values and hazard ratios (HR) with 95% confidence intervals (95% CI). Data were visualized as forest plots (using the “forestplot” R package) and survival curves.

We used KM plotter (http://kmplot.com/analysis/), to assess the relationships between *TGFBI* expression and prognosis in various cancers from the TCGA and GEO databases. Breast, gastric, lung, ovarian, and liver cancer datasets were each split into high- and low-expression subgroups using the “autoselect best cutoff” option, and *p* < 0.05 was considered significant.

### *TGFBI* Mutation Profiles

A comprehensive website cBioPortal (www.cbioportal.org) was used to explore, analyze, and visualize polydimensional cancer genomics data (Cerami et al., 2012). The *TGFBI* alteration frequencies, mutation types, and copy number alterations across the TCGA database were obtained using the “Cancer Types Summary” module of cBioPortal. To query the *TGFBI* genetic alteration characteristics, we set the “Quick select” field to “TCGA Pan Cancer Atlas Studies.”

The COSMIC (Catalog of Somatic Mutations in Cancer) website (https://cancer.sanger.ac.uk/cosmic/) is the largest and most comprehensive resource for exploring the impact of somatic mutations in human cancers (Tate et al., 2019). We used COSMIC to investigate the specific distribution of various *TGFBI* mutation types.

### Correlation Analyses

The Cancer Regulome Explorer (http://explorer.cancerregulome.org/) is a website that enables researchers to search, filter, and visualize analytical results obtained from the TCGA database. We used this website to investigate and visualize, at the chromosome level, the *TGFBI* mutation types in various cancer types, and the results were depicted in Circos plots. We set the pairwise correlation cutoff ≥0.4 and −log_10_ (*p* value) cutoff ≥10.

Pearson’s correlation analysis was performed to analyze the correlations between *TGFBI* expression and immunoinhibitory and immunostimulatory gene subsets, TMB, and MSI. The results were displayed as heatmaps basing on the “pheatmap” package in R.

### *TGFBI* Expression and Immune Cell Infiltration

We used the “Immune_Gene” module of TIMER2 to evaluate the correlations of *TGFBI* expression with immune cell infiltration across diverse cancer types in the TCGA database. The immune cells comprised CD4^+^ T cells, CD8^+^ T cells, regulatory T cells (Tregs), B cells, myeloid-derived suppressor cells (MDSCs), neutrophils, dendritic cells (DCs), monocytes, macrophages, mast cells, cancer-associated fibroblasts (CAFs), natural killer (NK) cells, endothelial cells, and T follicular helper (Tfh) cells.

### *TGFBI* Expression and TME Components

Tumor purity was assessed in 33 human cancers in the TCGA database basing on the “estimate” R package. Specifically, the ESTIMATE score is the sum of the immune and stromal scores, which represent the abundance of immune and stromal components, respectively (Yoshihara et al., 2013). Higher ESTIMATE scores correspond to lower tumor purity. Spearman’s correlation analysis was used to reveal the relationship between *TGFBI* expression and the immune, and stromal scores.

We also explored tumor RNA and DNA stemness scores (RNAss and DNAss) based on epigenetic and transcriptome data from the TCGA database. Specifically, RNAss is based on RNA-Seq data, and DNAss is based on DNA methylation data. Spearman’s correlation analysis was used to investigate the correlations of TGFBI expression with RNAss and DNAss.

### *TGFBI* Expression and Drug Responses

The CellMiner tool (https://discover.nci.nih.gov/cellminer/home.do) was employed to obtain National Cancer Institute (NCI)-60 data on *TGFBI* expression (i.e., transcript data) and drug responses (i.e., drug sensitivity based on data on the drug concentration that reduces total cell growth by 50% [GI50]). The NCI-60 data comprises molecular and pharmacological data on 60 diverse human cancer cell lines. The drug response data includes data on drugs approved by the US Food and Drug Administration and those assessed in clinical trials. Pearson’s correlation analysis was employed to evaluate the correlations between *TGFBI* expression and drug responses.

### Protein–Protein Interactions Network and Enrichment Analyses of TGFBI

The STRING (Search Tool for the Retrieval of Interacting Genes/Proteins) database (https://string-db.org/) contains known and predicted protein-protein interactions (PPIs) (Szklarczyk et al., 2019). We obtained the top 50 TGFBI-binding proteins using this database to construct a PPIs network. The parameters were set as follows: meaning of network edges: “evidence”; minimum required interaction score: “low confidence” (i.e., the line color represents the type of interaction evidence); and the maximum number of interactors to show: “no more than 50 interactors”. The PPIs network was visualized using STRING. Then the “Similar Gene Detection” module of GEPIA2 was used to obtain the top 100 TGFBI-correlated genes using TCGA data. Lastly, we conducted a Venn diagram analysis (http://jvenn.toulouse.inra.fr) to identify the common members of these two groups.

DAVID (Database for Annotation, Visualization, and Integrated Discovery, https://david.ncifcrf.gov/home.jsp; version 6.8) supplies a comprehensive, functional annotation tool to identify genes’ biological functions (Huang et al., 2009). It was used to conduct Kyoto Encyclopedia of Genes and Genomes (KEGG) and Gene Ontology (GO) enrichment analyses. The KEGG results were analyzed and visualized employing the “ggplot2” and “clusterProfiler” R packages. The GO biological process (BP), cellular component (CC), and molecular function (MF) results were obtained using the “cnetplots” R package.

### Statistical Analyses

*TGFBI* expression was compared between tumor and adjacent normal tissues employing the Oncomine database, with the results presented as *p* values, fold changes, and gene ranks. The survival results were presented as hazard ratios, 95% CI, and *p* values of log-rank tests. Using R version 4.0.4 (64-bit; https://www.r-project.org/) for the analyses. For all statistical analyses, *p* < 0.05 was considered statistically significant.

## Results

### TGFBI Expression Analysis in Pan-Cancer

**FIGURE 1:**
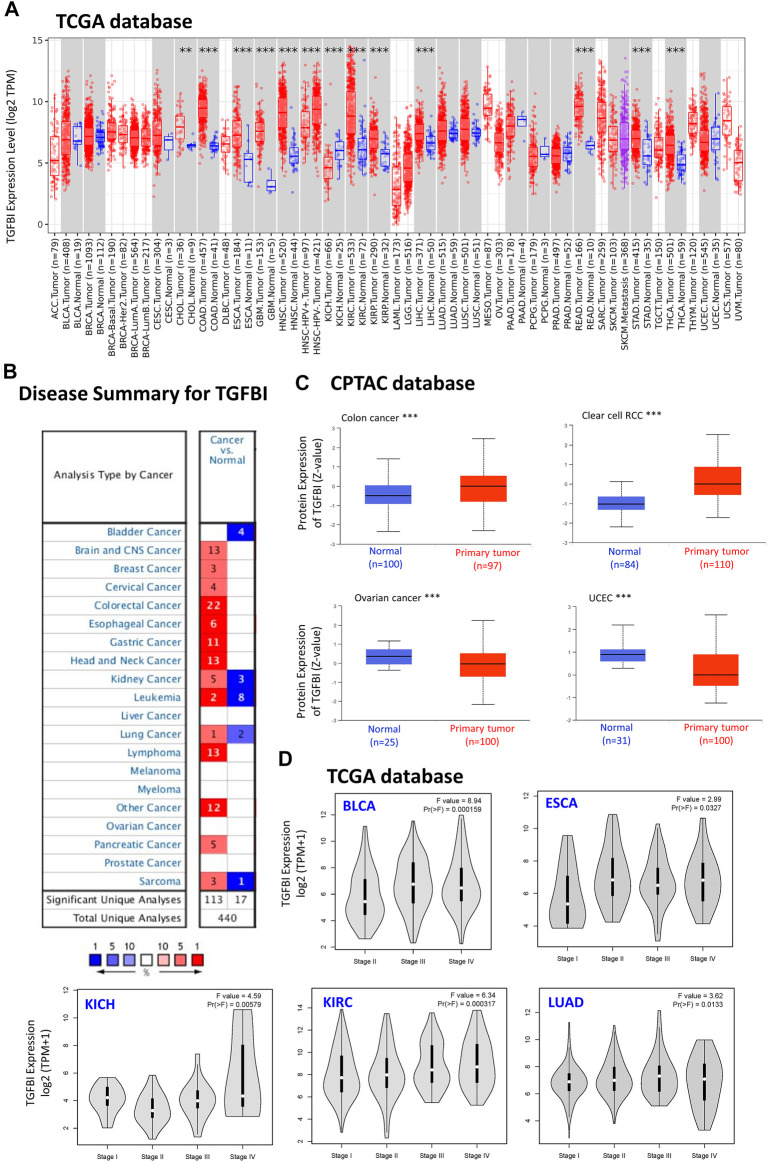
TGFBI expression in various types of human cancers and pathological stages. **(A)** Comparison of *TGFBI* expression between various cancers/cancer subtypes and normal tissues using TIMER2 based on TCGA data. *p < 0.05; **p < 0.01; ***p < 0.001. **(B)** Comparison of *TGFBI* expression between various cancer tissues and normal tissues in the Oncomine database. Blue represents low expression of TGFBI in the cancer tissues, red represents high expression, and gray represents no data. The number represents the number of studies that meet the filter criteria. **(C)** Comparison of TGFBI protein expression between primary colon cancer, clear cell renal cell carcinoma, uterine corpus endometrial carcinoma, and ovarian cancer tissues and normal tissues, based on CPTAC data. ***p < 0.001. **(D)** *TGFBI* expression in pathological stages I, II, III, and IV in BLCA, CESC, KICH, KIRC, and LUAD, based on TCGA data. The data were transformed using log2 (transcripts per million [TPM] + 1). F represents the statistical value of F test, Pr(>F) < 0.05 was considered significant.

To compare *TGFBI* expression between tumor and normal tissues, we applied TIMER2 to analyze *TGFBI* expression in various cancer types of TCGA. Compared to matched normal tissues, *TGFBI* was upregulated in cholangiocarcinoma (CHOL; *p* < 0.01), colon adenocarcinoma (COAD), esophageal carcinoma (ESCA), GBM, head and neck squamous cell carcinoma (HNSC), kidney renal clear cell carcinoma (KIRC), kidney renal papillary cell carcinoma (KIRP), liver hepatocellular carcinoma (LIHC), rectum adenocarcinoma (READ), stomach adenocarcinoma (STAD), and thyroid carcinoma (THCA; *p* < 0.001). However, *TGFBI* was downregulated in kidney chromophobe (KICH; *p* < 0.001; Figure 1A).

Furthermore, we applied the Oncomine database to compare *TGFBI* expression between tumor and matched normal tissues. *TGFBI* was upregulated in colorectal cancer, esophageal cancer, gastric cancer, head and neck cancer, and lymphoma (Figure 1B). In addition, we explored *TGFBI* expression across different tumor cell lines in the CCLE database. As shown in Supplementary Figure S1, RNAseq showed that *TGFBI* was upregulated in several cell lines, including kidney cancer, chondrosarcoma, upper aerodigestive tract cancer, glioma, osteosarcoma, thyroid cancer, liver cancer and mesothelioma cell lines.

We further evaluated the difference in TGFBI protein expression between the tumor tissues and normal tissues in the CPTAC database using UALCAN tools. TGFBI protein was upregulated in colon cancer and clear cell renal cell carcinoma compared to normal tissues (*p* < 0.001) but downregulated in OV and uterine corpus endometrial carcinoma (UCEC; *p* < 0.001; Figure 1C).

We also applied GEPIA2 to analyze *TGFBI* expression in various pathological stages of multiple cancer types. *TGFBI* expression was associated with bladder urothelial carcinoma (BLCA), ESCA, KICH, KIRC and lung adenocarcinoma (LUAD; all Pr(>F) < 0.05; Figure 1D). We also compared *TGFBI* expression between these TCGA tumor tissues and the corresponding normal tissues in the GTEx database, which showed that *TGFBI* was upregulated in 19 types of cancer tissues and downregulated in three types compared to normal tissues (Supplementary Figure S2).

**FIGURE 2:**
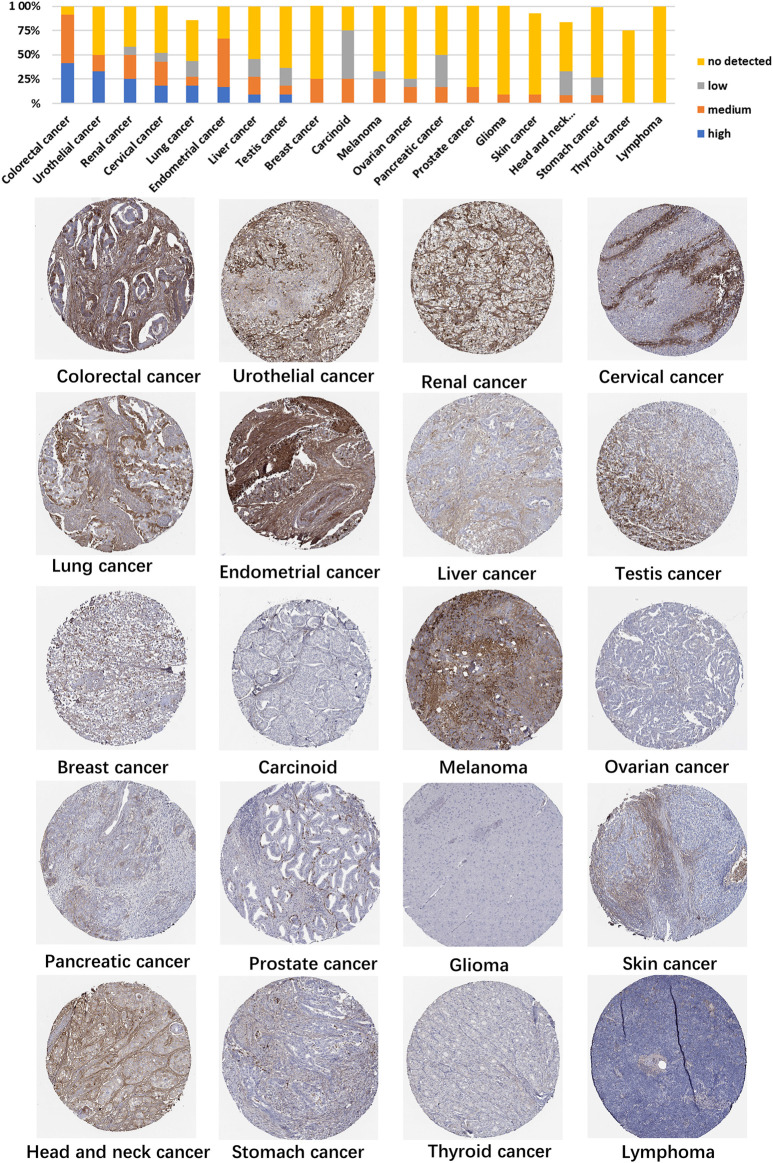
Representative immunohistochemical staining of TGFBI protein in various cancer tissues.

Using HPA data, we analyzed TGFBI protein expression. Analysis revealed that aberrant TGFBI protein expression was detected in 20 cancer types. The immunohistochemical results on TGFBI protein expression are shown in Figure 2.

### Correlation Between the Expression of *TGFBI* and Prognosis

**FIGURE 3:**
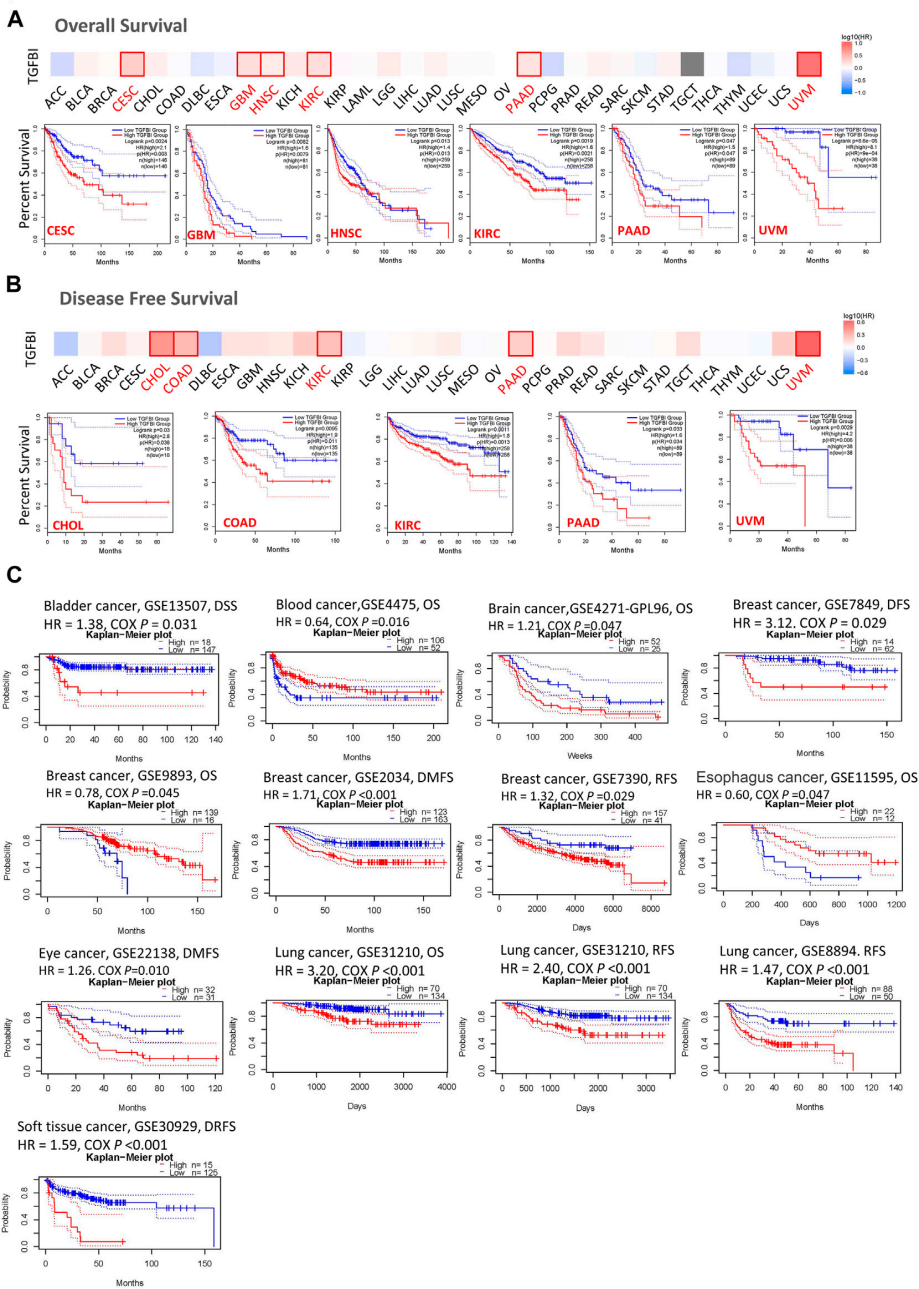
Associations of *TGFBI* expression with survival among cancer patients in the TCGA and GEO databases. *TGFBI* expression and **(A)** overall survival rate and **(B)** disease-free survival (DFS) rate in various tumors in TCGA were analyzed using GEPIA2. The Kaplan–Meier survival curves with significant results are shown. **(C)** Kaplan–Meier survival curves of TGFBI expression in 12 GEO cohorts (GSE13507, GSE4475, GSE4271, GSE7849, GSE9893, GSE 2034, GSE7390, GSE11595, GSE22138, GSE31210, GSE8894, and GSE30929), with significant differences shown. HR represents hazard ratios.

We assessed the associations between *TGFBI* expression and cancer survival outcomes in the TCGA database using GEPIA2. High *TGFBI* expression was associated with poor OS for cervical squamous cell carcinoma and endocervical adenocarcinoma (CESC, *p* = 0.0024), GBM (*p* = 0.0082), HNSC (*p* = 0.013), KIRC (*p* = 0.0019), pancreatic adenocarcinoma (PAAD; *p* = 0.047), and uveal melanoma (UVM; *p* < 0.001; Figure 3A). In addition, high *TGFBI* expression was associated with poor DFS for CHOL (*p* = 0.03), COAD (*p* = 0.0095), KIRC (*p* = 0.0011), PAAD (*p* = 0.033), and UVM (*p* < 0.0029; Figure 3B).

We also applied the PrognoScan database to analyze the associations between *TGFBI* expression and cancer survival outcomes, mainly based on GEO datasets (GSE13507, GSE4475, GSE4271, GSE7849, GSE9893, GSE2034, GSE7390, GSE11595, GSE22138, GSE31210, GSE8894, and GSE30929). High *TGFBI* expression was associated in Cox regression analyses with poor DSS in bladder cancer (*p* = 0.031), OS in brain cancer (*p* = 0.047), DFS, DMFS, and RFS in breast cancer (BRCA; all *p* < 0.05), DMFS in eye cancer (*p* = 0.010), OS and RFS in lung cancer (*p* < 0.001), and DRFS in soft tissue cancer (*p* < 0.001). In contrast, high *TGFBI* expression was associated with better OS in blood cancer (*p* = 0.016), breast cancer (*p* = 0.045), and esophageal cancer (*p* = 0.047; Figure 3C).

**FIGURE 4:**
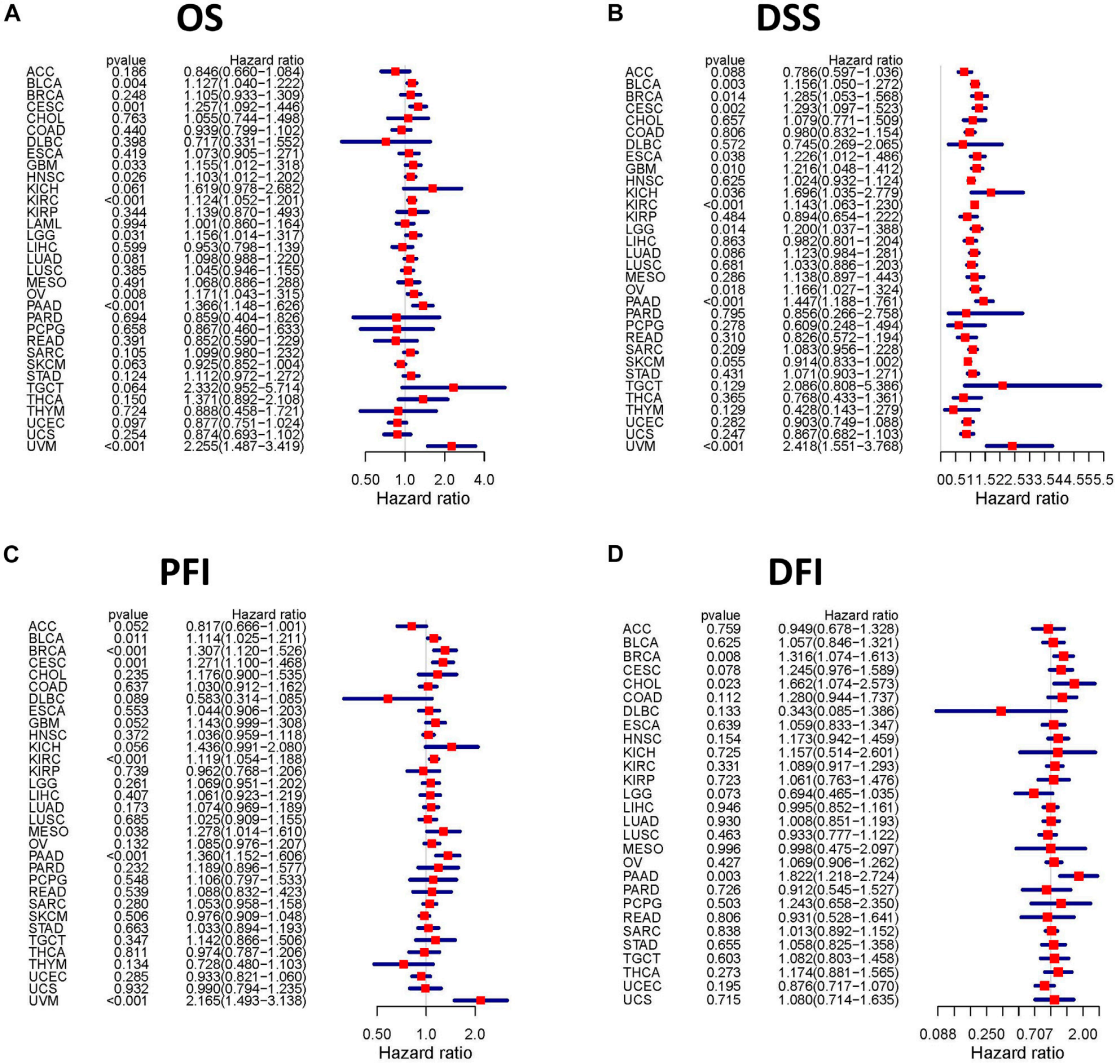
Correlation analysis of *TGFBI* expression and patient survival by the Cox regression analysis in different cancer types. HR < 1 represents low risk and HR > 1 represents high risk. Univariate Cox proportional hazard regression models were applied for the association tests.

**FIGURE 5:**
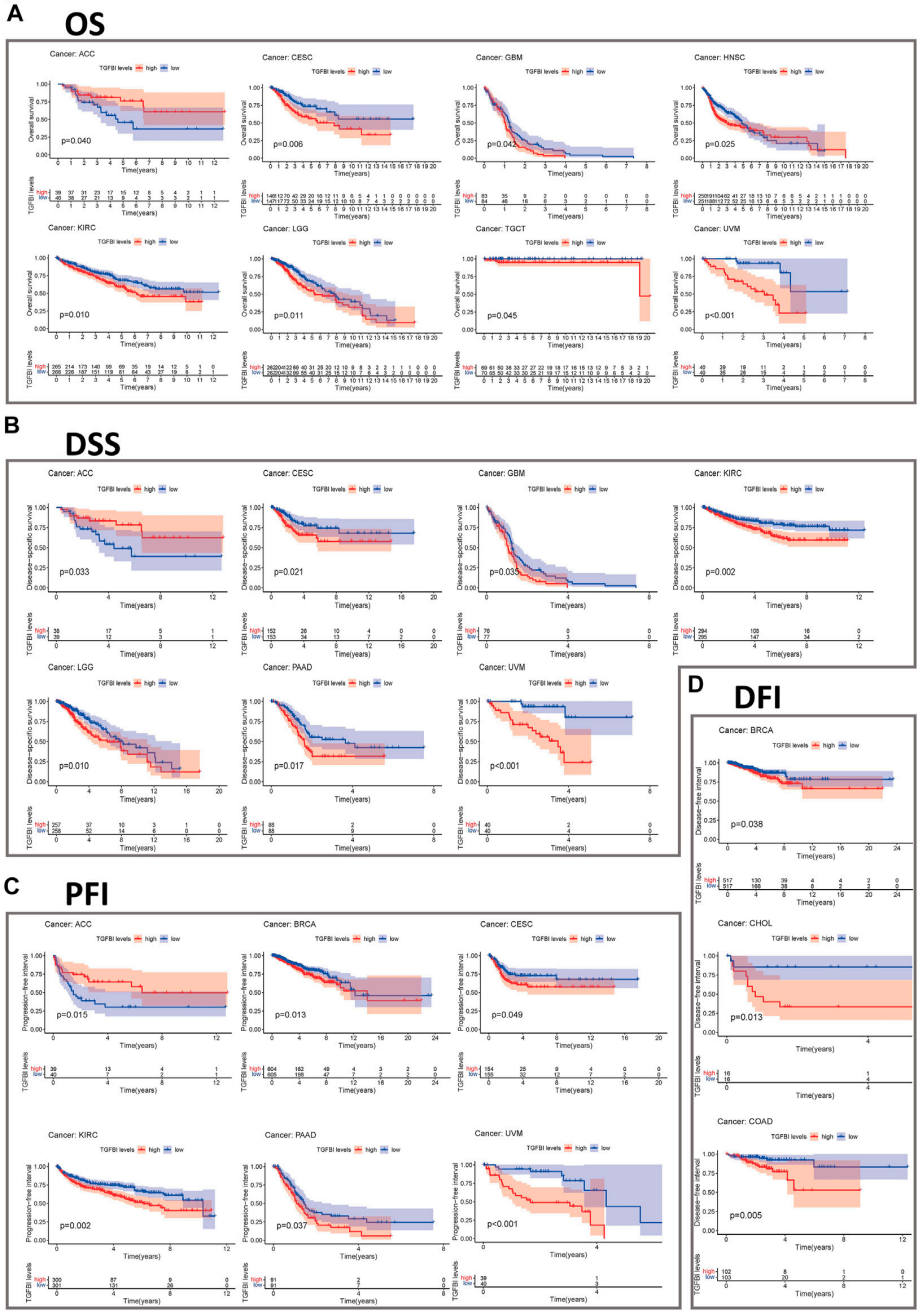
Kaplan–Meier survival curves comparing pan-cancer high and low expression of *TGFBI*. **(A)** OS survival curves for *TGFBI* in different cancers; **(B)** DSS survival curves for *TGFBI* in different cancers; **(C)** PFI survival curves for *TGFBI* in different cancers; **(D)** DFI survival curves for *TGFBI* in different cancers.

We assessed the correlation between the expression of *TGFBI* and OS, DSS, PFI, and DFI in different types of cancer using a single-variate Cox regression analysis based on TCGA database. The results are summarized in forest plot (Figure 4). KM analysis showed that high *TGFBI* expression predicted poor prognosis of CESC, GBM, KIRC, LGG, testicular germ cell tumors (TGCT), and UVM (all *p* < 0.05) but good prognosis of adrenocortical carcinoma (ACC) and HNSC (all *p* < 0.05; Figure 5A). In addition, increased expression of *TGFBI* was associated with poor DSS in CESC, GBM, KIRC, LGG, PAAD, and UVM, whereas, increased *TGFBI* expression predicted good DSS in ACC (all *p* < 0.05, Figure 5B). The same method is used to analyze PFI in 33 TCGA tumors. *TGFBI* had a protective role in ACC. On the other hand, *TGFBI* had a detrimental role in BRCA, CESC, KIRC, PAAD, and UVM (all *p* < 0.05, Figure 5C). Finally, we also analyzed the DFI in 33 TCGA tumors. *TGFBI* expression had a detrimental role in BRCA, CHOL, and COAD (all *p* < 0.05, Figure 5D).

Then we studied the prognostic value of *TGFBI* expression was further evaluated using KM plotter. Notably, high *TGFBI* expression was significantly associated with poor DMFS, OS, post-progression survival (PPS), and RFS in breast cancer (*p* < 0.01; Supplementary Figure S3A). However, high *TGFBI* expression was associated with good DSS, OS, progression-free survival (PFS), and RFS in liver cancer (*p* < 0.05; Supplementary Figure S3B). High *TGFBI* expression was associated with poor first progression (FP), OS, and PPS in gastric cancer (*p* < 0.001; Supplementary Figure S3C). High *TGFBI* expression was associated with poor FP and OS but good PPS in lung cancer (*p* < 0.001; Supplementary Figure S3D). Moreover, high *TGFBI* expression was associated with poor OS, PFS, and PPS in OV (*p* < 0.05; Supplementary Figure S3E).

### The Mutation Profiles of *TGFBI* in Pan-Cancer

**FIGURE 6:**
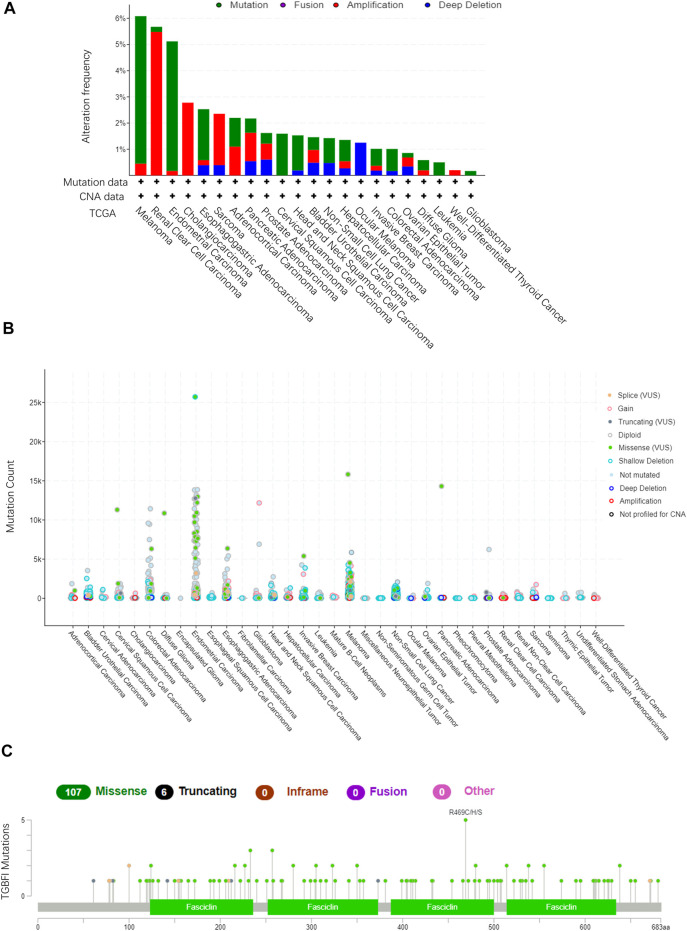
*TGFBI* mutation landscape in various cancer types. **(A)** *TGFBI* mutation frequency in various TCGA pan-cancer studies according to cBioPortal. **(B)** *TGFBI* mutation count in various TCGA cancer types according to cBioPortal. **(C)** TGFBI mutations across protein domains in various cancer types.

Using TCGA data in cBioPortal (10,967 samples from 32 studies), we assessed the *TGFBI* alteration frequency and mutation count in tumor samples. Melanoma had the highest frequent *TGFBI* alteration (>6%), with “mutation” as the primary type of alteration (Figures 6A,B). Data on the *TGFBI* alteration types, sites, and numbers of cases, including data on missense, nonsense (truncation), inframe, and fusion mutations, are shown in Figure 6C. The *TGFBI* mutation hotspot was R469C/H/S (missense mutation) in the FAS1 domain, which occurred in four cancers (UCEC, CESC, COAD, and skin cutaneous melanoma [SKCM]) in four patients.

Moreover, we explored the impact of somatic *TGFBI* mutations in human cancer. Using the COSMIC website, nonsense (truncation) mutations were found in lung cancer (10%); missense mutations were found in endometrial (7.14%), hematopoietic and lymphoid (40%), large intestine (15%), lung (20%), and skin (61.54%) cancer; and synonymous substitutions were found in breast (28.57%), endometrial (28.57%), and large intestine (10%) cancer. G > A was the primary mutation type in these cancer cases (Supplementary Figure S4).

### Genomic Analysis of *TGFBI* in Cancer

Using the Cancer Regulome Explorer website, we investigated the relevant genomic correlation between *TGFBI* gene and certain signatures. DNA methylation, somatic copy number, somatic mutation, microRNA expression, and protein expression were showed to reveal the interrelation in different tumors using data from the TCGA database. The *TGFBI* was associated with above signatures in ACC, BLCA, BRCA, ESCA, STAD, GBM, HNSC, KIRC, LUAD, OV, SKCM, THCA, UCEC, lung squamous cell carcinoma (LUSC), and prostate adenocarcinoma (PRAD) (Supplementary Figure S5).

### *TGFBI* Expression and Immune Cell Infiltration

TGFBI is involved in immune cell infiltration and inflammatory responses, which play key roles in cancer initiation, progression, and metastasis. To investigate immune cell infiltration at the pan-cancer level, we used TIMER2.0 to explore the associations between *TGFBI* expression in human cancer and the infiltration of various types of immune cells (based on CIBERSORT, CIBERSORT-ABS, XCELL, MCPCOUNTER, QUANTISEQ, and EPIC algorithms). Overall, *TGFBI* expression was positively correlated with neutrophils, monocytes, macrophages, CAFs and MDSCs, and negatively correlated with B cells, T follicular helper (Tfh) cells, and CD8^+^ T cells. Thus, *TGFBI* expression may play vital roles in the immune cell infiltration process. The immune cell infiltration was quite different in OV, PRAD, UVM, and THCA due to the distinct TME components in the central nervous system (Supplementary Figure S6).

### Correlations of *TGFBI* Expression with Immunomodulatory Genes, TMB, and MSI

**FIGURE 7:**
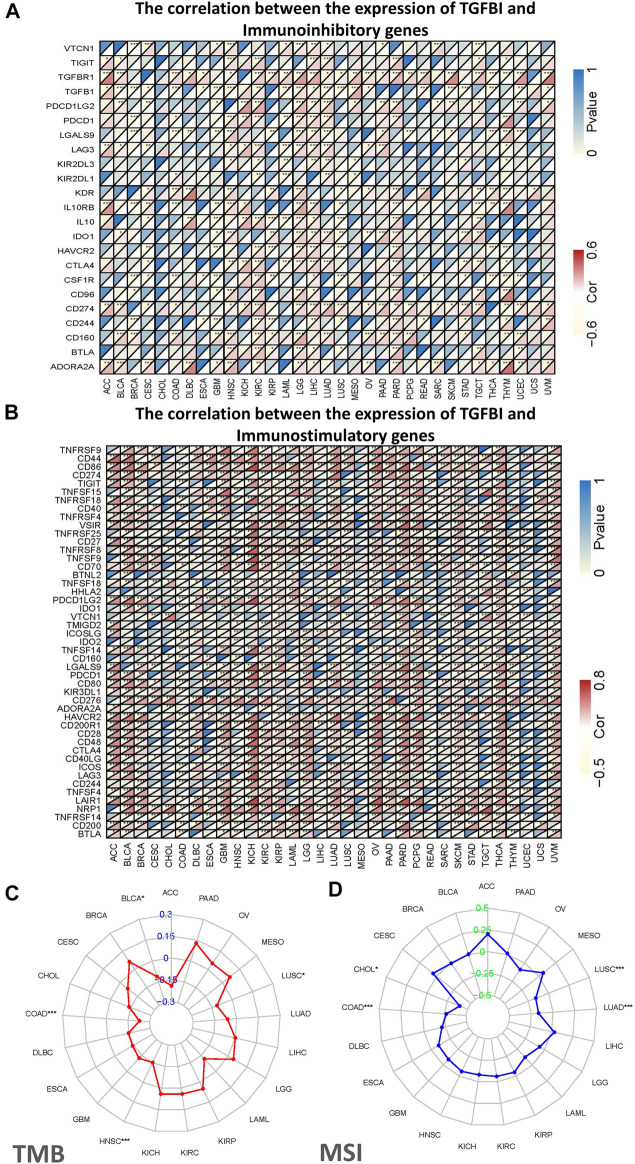
Correlations of *TGFBI* expression with immunomodulatory genes, TMB, and MSI in various cancer types. Correlations of *TGFBI* expression with **(A)** immunoinhibitory and **(B)** immunostimulatory genes. For each pair, the upper left triangle is colored to represent the *p* value, and the lower right one is colored to indicate Spearman’s correlation coefficient. **(C)** Radar chart of the overlap between TMB and *TGFBI* expression. The blue number represents Spearman’s correlation coefficient. **(D)** Radar chart of the overlap between MSI and *TGFBI* expression. The green number represents Spearman’s correlation coefficient. *p < 0.05, **p < 0.01, ***p < 0.001.

Tumor immunotherapy is a novel therapeutic strategy which been proven efficacious in multiple types of cancers. Thus, we explore whether TGFBI could be used as a novel target for tumor immunotherapy. As shown in Figure 7A, in most cancers, except CHOL, ESCA, sarcoma (SARC), SKCM and UCS, *TGFBI* expression was significantly correlated with immunoinhibitory genes. Additionally, except CESC, CHOL, mesothelioma (MESO), READ, SARC, UCEC and UCS, *TGFBI* expression was significantly correlated with immunostimulatory genes (Figure 7B).

We also explored the associations of *TGFBI* expression with TMB and MSI in various types of cancer. The *TGFBI* expression was negatively correlated with TMB in COAD, HNSC, LUSC and BLCA (all *p* < 0.05; Figure 7C). In addition, *TGFBI* expression was negatively correlated with MSI in LUSC, LUAD, COAD, and CHOL (all *p* < 0.05; Figure 7D).

### *TGFBI* Expression and TME Components

**FIGURE 8:**
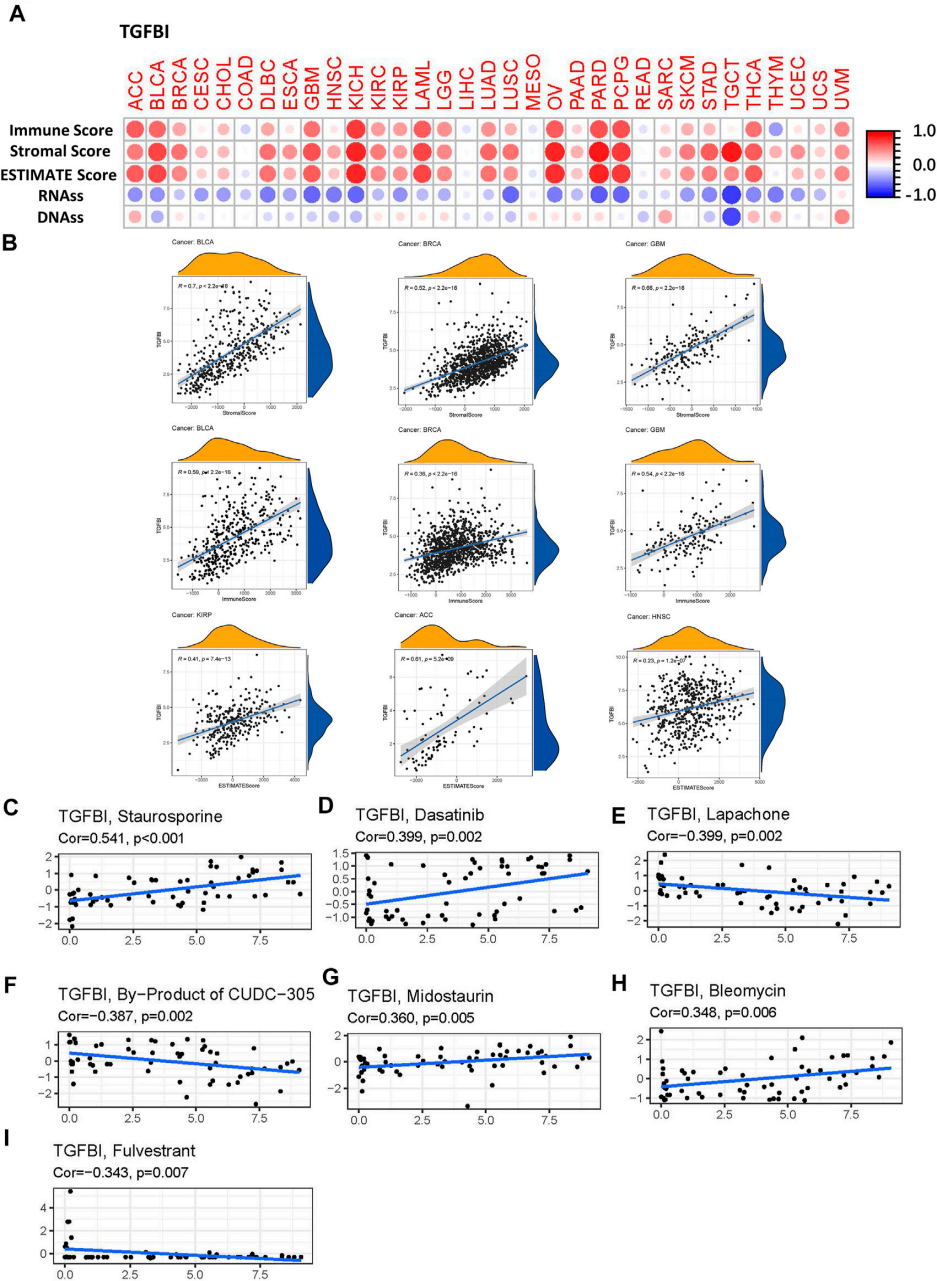
Correlation matrix of *TGFBI* expression with tumor microenvironment (TME) components, stemness scores, and drug sensitivity in a pan-cancer analysis. **(A)** *TGFBI* expression was correlated with ESTIMATE score, immune score, stromal score, RNAss, and DNAss in various cancers. Red and blue dots indicate a positive and negative correlation, respectively, between *TGFBI* expression in cancer and immune/stromal score. **(B)** Top three scatter plots of correlation between *TGFBI* and stromal score, immune score, ESTIMATE score in various cancers. *TGFBI* expression was positively associated with **(C)** staurosporine, **(D)** dasatinib, **(G)** midostaurin, and **(H)** bleomycin sensitivity, and negatively associated with **(E)** lapachone, **(F)** CUDC-305 byproduct, and **(I)** fulvestrant sensitivity. R represents the correlation coefficient, Cor represents correlation.

Since our results have confirmed the immunoregulation role of the TGFBI in various types of cancer, it is vary needed to explore further the correlation between TGFBI expression and TME. We explored the correlations of *TGFBI* expression with TME components, using the ESTIMATE algorithm to calculate the ESTIMATE, stromal, and immune scores in diverse tumor types in the TCGA database. *TGFBI* expression was positively associated with stromal and immune scores in the pan-cancer analysis (all *p* < 0.05; Figures 8A,B). Furthermore, we assessed the correlations between *TGFBI* expression and tumor stemness in a pan-cancer analysis using TCGA data. *TGFBI* expression was negatively correlated with RNAss, and positively correlated with DNAss, in ACC, SARC, THCA, thymoma (THYM) and UVM (Figure 8A).

### The Drug Responses Analysis of *TGFBI* Expression

To investigate the potential relationships between *TGFBI* expression and drug responses in various types of human cancer, we performed a correlation analysis to identify potential drug candidates using CellMiner. *TGFBI* expression was positively correlated with staurosporine, dasatinib, midostaurin, and bleomycin sensitivity (all *p* < 0.05; Figures 8C,D,G,H), but negatively associated with lapachone, CUDC-305 byproduct, and fulvestrant sensitivity (all *p* < 0.05; Figures 8E,F,I).

### Protein-Protein Interactions Network and Enrichment Analyses of TGFBI

**FIGURE 9:**
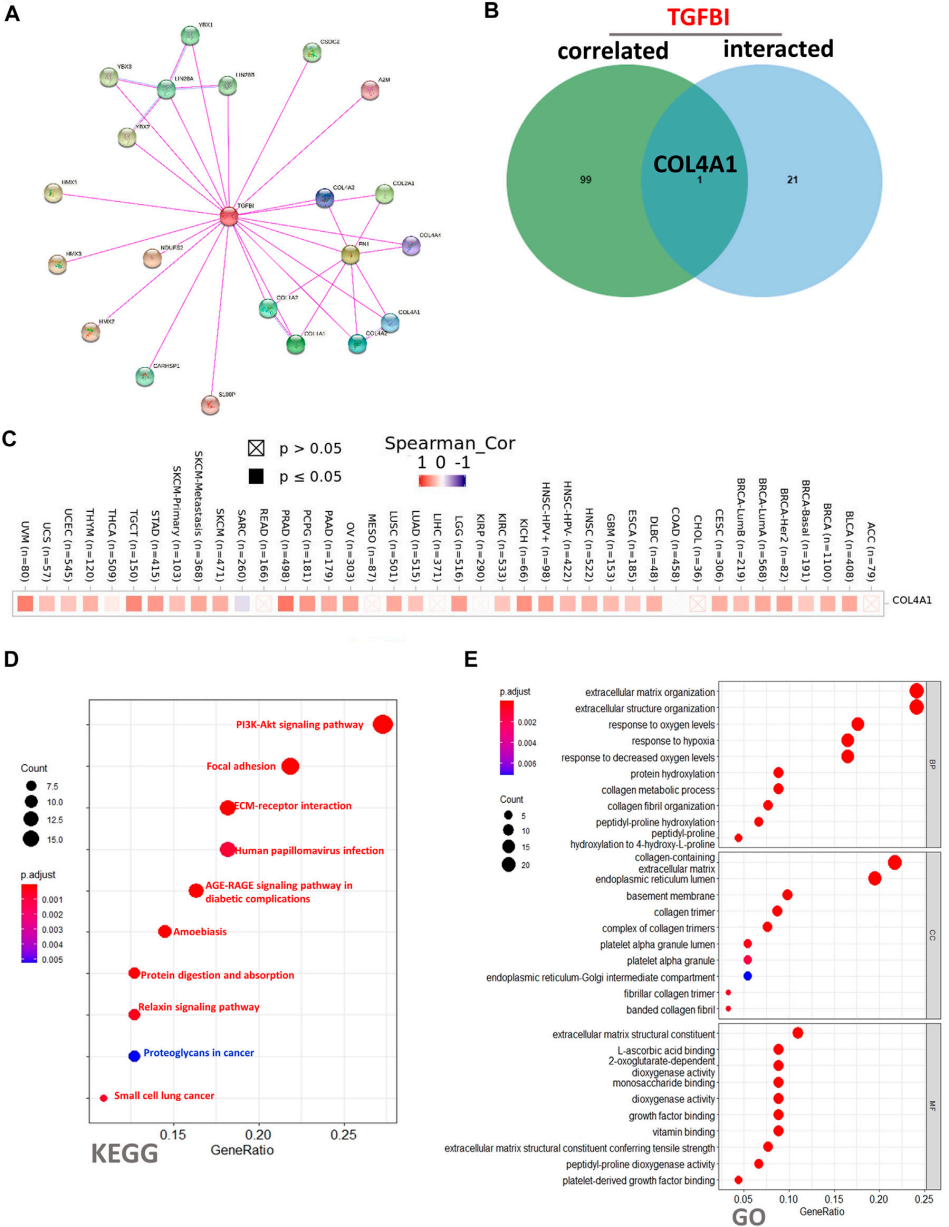
TGFBI-related gene enrichment analysis. **(A)** Experimentally determined TGFBI-binding proteins, based on STRING. **(B)** Venn diagram analysis of TGFBI-binding proteins (based on STRING) and TGFBI-correlated genes (based on GEPIA2). **(C)** Corresponding heatmap in various cancer types. **(D)** KEGG pathway enrichment analysis and **(E)** Gene Ontology (GO) biological process, cellular component, and molecular function enrichment analysis of TGFBI-binding proteins (based on STRING) and TGFBI-correlated genes (based on GEPIA2).

To clarify the molecular mechanisms of TGFBI in tumorigenesis, we conducted a PPIs network analysis of TGFBI and enrichment analyses of TGFBI-binding proteins (based on STRING) that were also TGFBI-correlated genes (based on GEPIA2). The PPIs network, constructed using STRING, was in the base of experimental evidence and had 22 nodes and 34 edges (Figure 9A). We obtained 50 TGFBI-binding proteins supported by experimental evidence in STRING. Using GEPIA2, we then obtained the top 100 genes associated with *TGFBI* expression. Next, a Venn diagram analysis of these two groups showed one common member, namely, *COL4A1* (Figure 9B). The heatmap in Figure 9C shows the positive correlation between *TGFBI* and *COL4A1* expression.

To conduct KEGG and GO functional enrichment analyses of TGFBI, we used DAVID 6.8. The KEGG analysis showed that the “PI3K-Akt signaling pathway”, “Focal adhesion”, and “ECM–receptor interaction” might be involved in the effects of TGFBI on tumor pathogenesis (Figure 9D). The GO analysis showed similar results, as TGFBI was closely involved in a variety of terms, such as “extracellular matrix organization” in the BP category, “collagen-containing extracellular matrix” in the CC category, and “extracellular matrix structural constituent” in the MF category (Figure 9E).

## Discussion

In recent years, a number of studies have shown that TGFBI is closely related to tumor growth, invasion, metastasis, and drug responses. However, there are no previous pan-cancer studies on the relationship between *TGFBI* expression and various tumors. In this study, we investigated the *TGFBI* expression, survival status, *TGFBI* mutations, immune cell infiltration, and associated molecular pathways, to explore the potential cellular mechanisms of TGFBI in various types of cancers.

Based on the Oncomine and TIMER2.0 databases, *TGFBI* was upregulated in CHOL, COAD, ESCA, GBM, HNSC, KIRC, KIRP, LIHC, READ, STAD, and THCA, and downregulated in KICH, compared to adjacent normal tissues. A previous study also found that *TGFBI* was upregulated in colon cancer, promoting metastasis (Ma et al., 2008). Another study showed that the proliferation and metastasis ability of oral squamous cell carcinoma cells was also enhanced by *TGFBI* upregulation (Wang et al., 2019).

We also identified associations between *TGFBI* expression and prognosis of lung squamous cell, gastric, colorectal, and bladder cancer, which concurs with previous studies (Pajares et al., 2014; Suzuki et al., 2018; Zhang et al., 2019; Zou et al., 2019; Yang et al., 2020). Overall, *TGFBI* was upregulated in most tumors, though the *TGFBI* expression and survival analysis suggested different conclusions for different tumors. For example, *TGFBI* upregulation had a detrimental effect on UVM, which was consistent with a previous study showing that human metastatic melanoma express and released a significantly higher amount of TGFBI (Lauden et al., 2014), rendering TGFBI a potential target for therapeutic interventions. We also found some contradictory results about the prognostic value of *TGFBI* expression in LUAD and STAD. High *TGFBI* expression was significantly associated with poor OS in LUAD and STAD in the KM plotter analysis but not in the GEPIA2 analysis. These inconsistent results may be attributable to the different data collection methods, types of cancer patients, or biological characteristics of each sample. These finding suggest that TGFBI might be a novel prognostic biomarker for some types of cancer.

Another essential finding in this study was that *TGFBI* expression was correlated with different levels of immune cell infiltration among diverse types of tumors (Du et al., 2020; Nakazawa et al., 2020). *TGFBI* expression was positively correlated with CAFs, macrophages, monocytes, MDSCs, and neutrophils, and negatively correlated with B cells, Tfh cells, and CD8^+^ T cells. In this study, *TGFBI* expression was an indicator of the level of infiltration of CAFs, which represent the main component of the tumor stroma and are strongly associated with epithelial–mesenchymal transition, massive stromal cell infiltration, and poor cancer prognosis. CAF infiltration can be used to predict survival in patients with oral squamous cell carcinoma (Ko et al., 2020). Tumor-associated macrophages promote OV cell migration, adhesion, and invasion by secreting TGFBI, and they have been associated with short PFS in high-grade serous OV patients (Steitz et al., 2020). Although we found that *TGFBI* expression was negatively correlated with CD8^+^ T cells, another study showed that both high stromal *TGFBI* expression and intratumoral CD8^+^ T cells infiltration were associated with poor prognosis and drug resistance in lung cancer patients (Nakazawa et al., 2020). These contradictory findings necessitate further research.

In this study, we presented evidence regarding the correlations between *TGFBI* expression and MSI and TMB across the TCGA pan-cancer. MSI is a molecular marker of deficient mismatch repair (MMR), which leads to errors in DNA replication, the accumulation of DNA mutations, and a high TMB in many cancer types (Baretti and Le, 2018). TMB is an emerging marker for identifying potential responders to immunoinhibitory and immunostimulatory factors across cancer types (Goodman et al., 2017). The *TGFBI* expression was negatively correlated with TMB in COAD, HNSC, BLCA, and LUSC. In addition, *TGFBI* expression was negatively correlated with MSI in LUSC, LUAD, COAD, and CHOL. Our results suggest that the relationships of *TGFBI* expression with TMB and MSI are diverse in those types of cancer. *TGFBI* might influence tumorigenesis in these cancer types by participating in the process of genetic alterations. expression might also independently predict responses to immunoinhibitory and immunostimulatory factors.

In addition, we explored the relationships of *TGFBI* expression with TME components and tumor stemness. The TME plays an important role in tumorigenesis and metastasis (Binnewies et al., 2018; Yan et al., 2019; Baghban et al., 2020). Based on the ESTIMATE algorithm and TCGA data, *TGFBI* expression was correlated with the levels of immune and stromal cell infiltration. Moreover, we analyzed the correlations between *TGFBI* and tumor stemness scores (RNAss, and DNAss), which are associated with tumor pathology and tumor dedifferentiation (Malta et al., 2018). The tumor stemness analysis of the pan-cancer TCGA data showed that RNAss and DNAss was negatively and positively correlated with prognosis. Similarly, a previous study indicated that TGFBI may support cancer stem cell growth and tumor progression to metastasis in breast cancer (Fico and Santamaria-Martínez, 2020). Besides, we analyze the correlation between *TGFBI* expression with various immune cell infiltration in different types of human cancers. Overall, *TGFBI* expression showed a significant was positively correlated correlation with immune cell infiltrating levels of multiple infiltrates including CD8^+^ T cells, dendritic cells, macrophages, monocytes, NK cells, neutrophils, Tregs, and Tfh. Otherwise, MDSCs abundance was negatively correlated with *TGFBI* expression. The profile indicated that *TGFBI* play an important role in the recruitment and regulation of immune infiltrating cells in tumors. It is worth noting that in tumors such as ACC, BLCA, BRCA, HNSC, DLBC, GBM, LGG, THCA, THYM, and UVM (Supplementary Figure S7), the correlation between *TGFBI* expression and immune cell infiltration was subtly different, which may be caused by the various immune cell infiltration ratios in different types of cancers. Our results emphasize that *TGFBI* expression is closely related to tumor cells, immune cell infiltration, and TME components, affecting cancer prognosis. These results provide new insights for developing more effective treatment.

Additionally, we assessed the correlations between *TGFBI* expression and drug response in various human cancer cell lines from CellMiner database, which facilitates the integration and study of molecular and pharmacological data on the 60 different human cancer cell lines. *TGFBI* expression was positively correlated with staurosporine, dasatinib, midostaurin, and bleomycin sensitivity, but negatively associated with lapachone, CUDC-305 byproduct, and fulvestrant sensitivity. A previous study reported that *TGFBI* is frequently methylated, the loss of *TGFBI* is associated with paclitaxel resistance in OV (Wang et al., 2012), but the overexpression of *TGFBI* makes nasopharyngeal carcinoma cells sensitive to cisplatin (Bissey et al., 2018), and increase the sensitivity of human non-small cell lung cancer cell lines to etoposide, paclitaxel, cisplatin and gemcitabine (Irigoyen et al., 2010). These studies suggest that *TGFBI* might be used as a predictive factor of chemotherapy in some tumors (Yin et al., 2020).

Furthermore, we predicted the PPIs network and KEGG pathways associated with TGFBI. TGFBI was involved in “PI3K/Akt signaling pathway,” “ECM–receptor interaction” and “Focal adhesion,” which is consistent with the current research on TGFBI. Previous studies have reported that TGFBI play as irreplaceable role in inducing suppressive mesothelioma tumorigenesis and progression through the PI3K/Akt signaling pathway (Wen et al., 2011b). The ECM has essential roles in tumorigenesis and cancer progression, and different ECM components depend on different signaling mechanisms in various cancer cells. In addition, TGFBI preferentially interacts via a ß3 integrin-receptor-mediated mechanism to modulate paclitaxel-induced OV cell death (Tumbarello et al., 2012). In addition, TGFBI activates the focal adhesion kinase signaling pathway by binding to integrin *α*V*β*5, significantly enhancing the invasion of pancreatic ductal adenocarcinoma (Costanza et al., 2019). Our results indicated that TGFBI is an oncogenic ECM formation protein that may serve as a valuable therapeutic target for new anti-cancer treatment strategies.

There are several limitations in this study. First, although the study involved a bioinformatic analysis of *TGFBI*, including its expression, prognostic value, associations with immune cell infiltration, and mutation status in various human cancer types, there were no *in vivo* or *in vitro* experiments to validate the results. Therefore, future studies should focus on the mechanisms of TGFBI in various human cancer types. Second, we analyzed prognostic data from TCGA, KM plotter, and PrognoScan, and there might be heterogeneity among these datasets. So, higher-resolution analysis such as single-cell RNA sequencing should be performed to verify our claims.

In conclusion, we investigated *TGFBI* expression characteristics, prognostic value, mutation profiles, associations with tumor-infiltrating immune cells, and associated molecular pathways in various types of cancer. We have provided new clues for improving cancer diagnosis and developing cancer immunotherapies that target *TGFBI*.

## Data Availability

The original contributions presented in the study are included in the article/Supplementary Material, further inquiries can be directed to the corresponding author.
