# Supplementary material for: Pan-Cancer Analysis of the Associations of TGFBI Expression With Prognosis and Immune Characteristics
Source: Front Mol Biosci. 2021 Oct 4;8:745649. doi: 10.3389/fmolb.2021.745649 (PMC8521171; doi:10.3389/fmolb.2021.745649)
Supplement: Supplementary file 1 [file DataSheet1.docx]

Supplementary Material

##
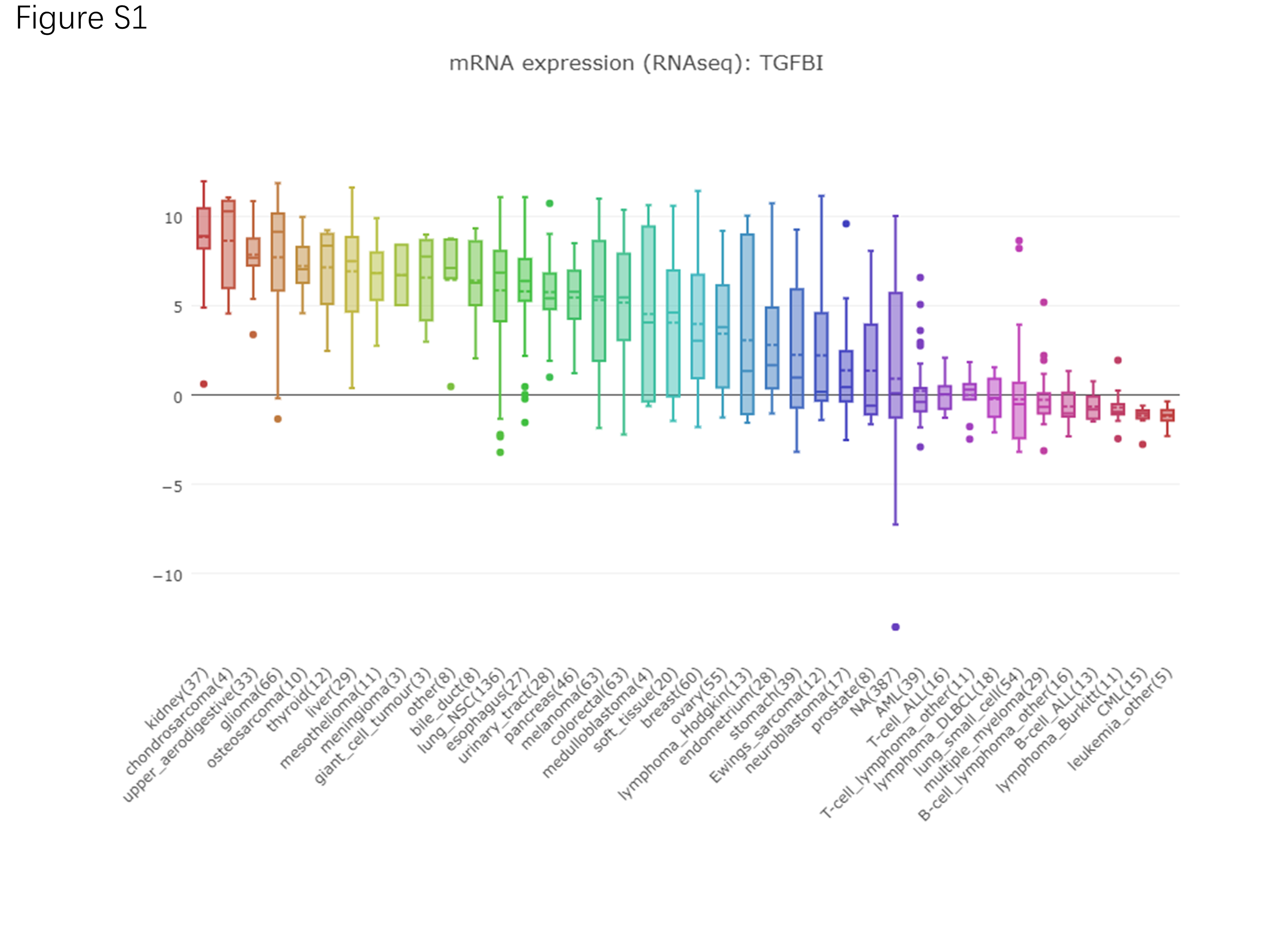
Supplementary Figures

## Supplementary Figure S1. Differential *TGFBI* expression in tumor cell lines.


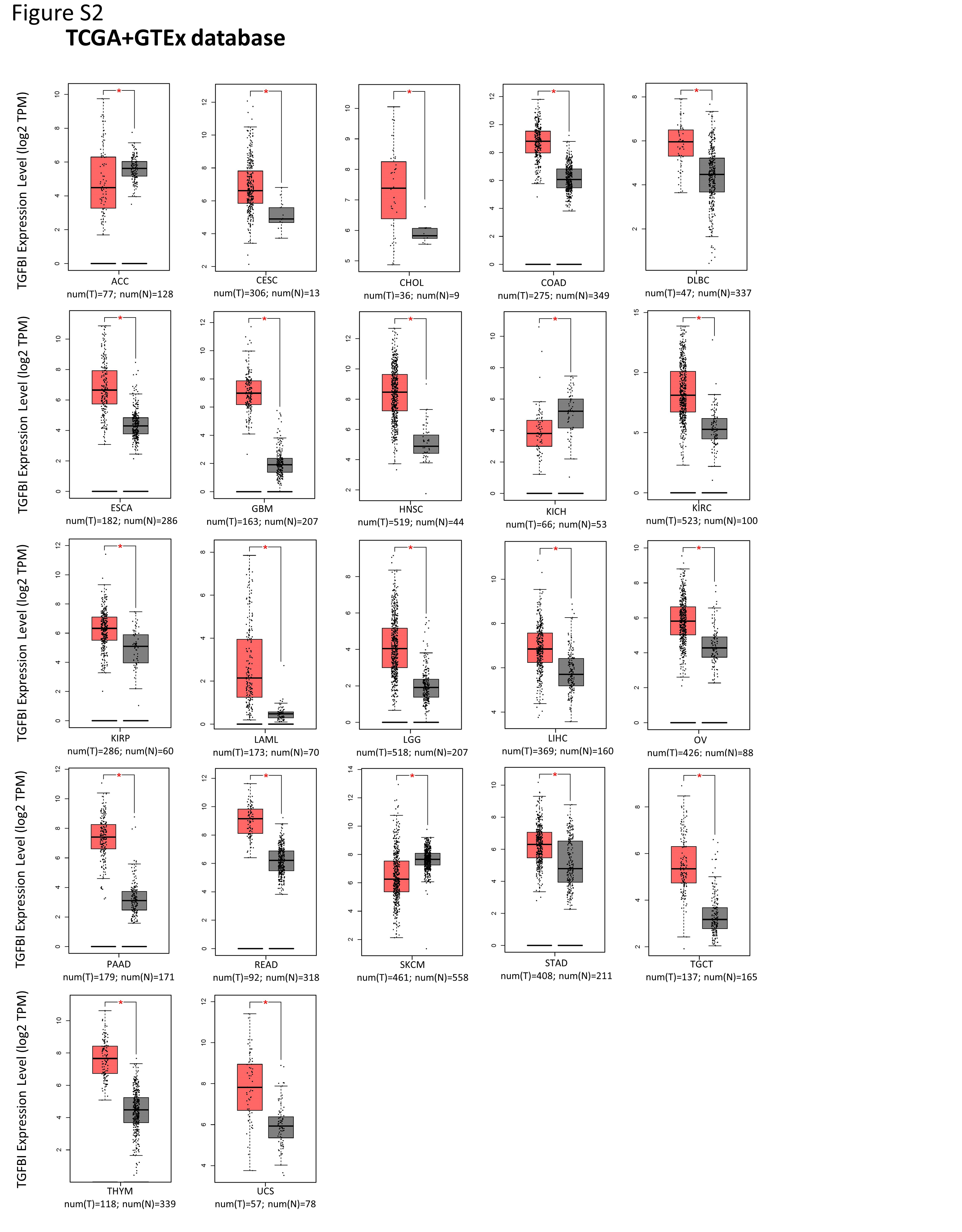


**Supplementary Figure S2.** Box plots of *TGFBI* expression in cervical squamous cell carcinoma and endocervical adenocarcinoma (CESC), cholangiocarcinoma (CHOL), colon adenocarcinoma (COAD), diffuse large B-cell lymphoma (DLBC), glioblastoma multiforme (GBM), head and neck squamous cell carcinoma (HNSC), kidney chromophobe (KICH), kidney renal clear cell carcinoma (KIRC), acute myeloid leukemia (LAML), low-grade glioma (LGG), liver hepatocellular carcinoma (LIHC), ovarian cancer (OV), rectum adenocarcinoma (READ), skin cutaneous melanoma (SKCM), stomach adenocarcinoma (STAD), testicular germ cell tumor, and uterine carcinosarcoma (UCS) in the TCGA database, with corresponding GTEx normal tissues used as controls. **P*<0.05.

**
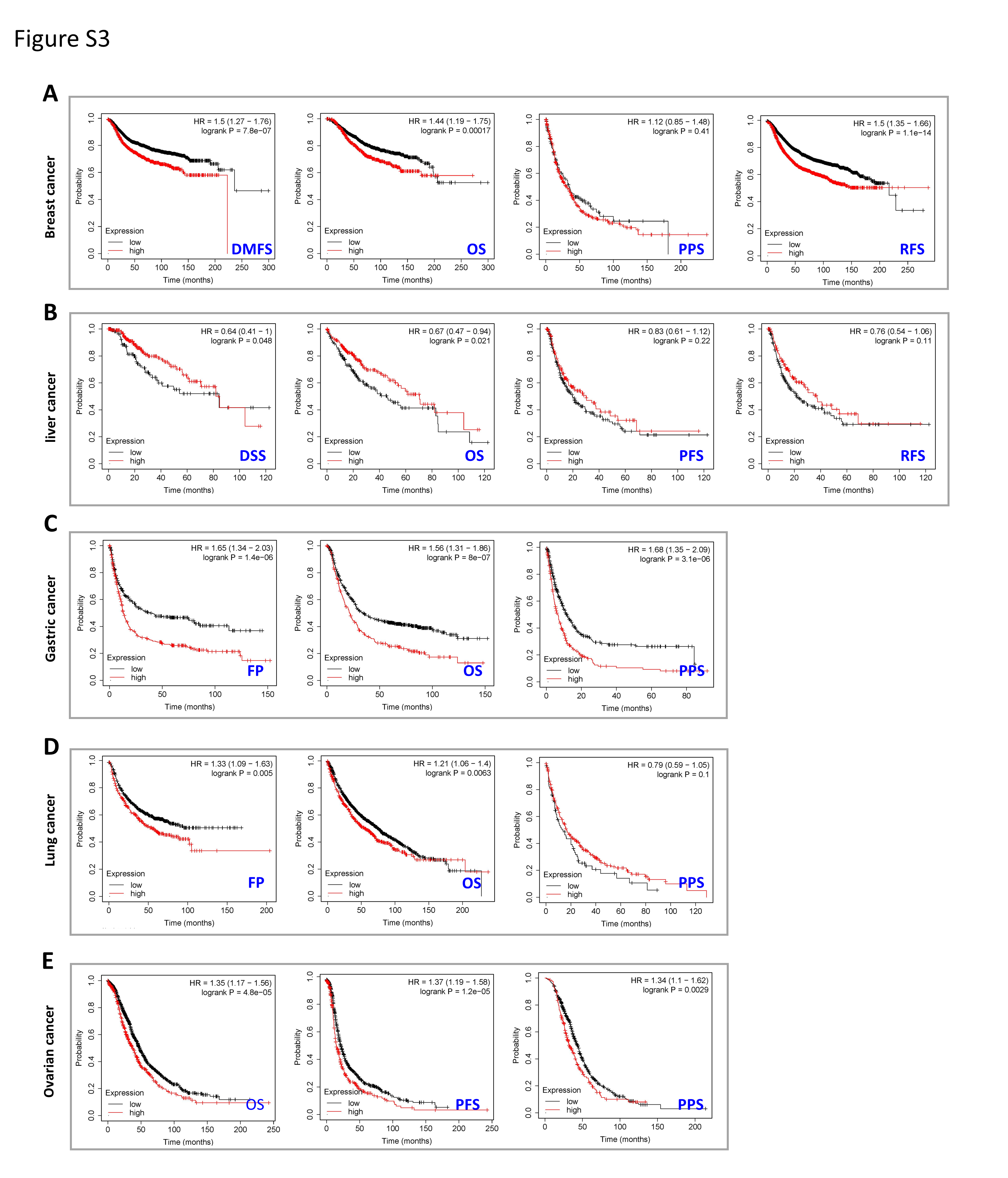
**

**Supplementary Figure S3.** Associations of *TGFBI* expression with cancer prognoses using Kaplan–Meier (KM) analysis. Analyses of TGFBI expression and DMFS, OS, PPS, RFS, PFS, DSS, and FP in (A) breast, (B) liver, (C) gastric, (D) lung, and (E) ovarian cancer using KM plotter.

**
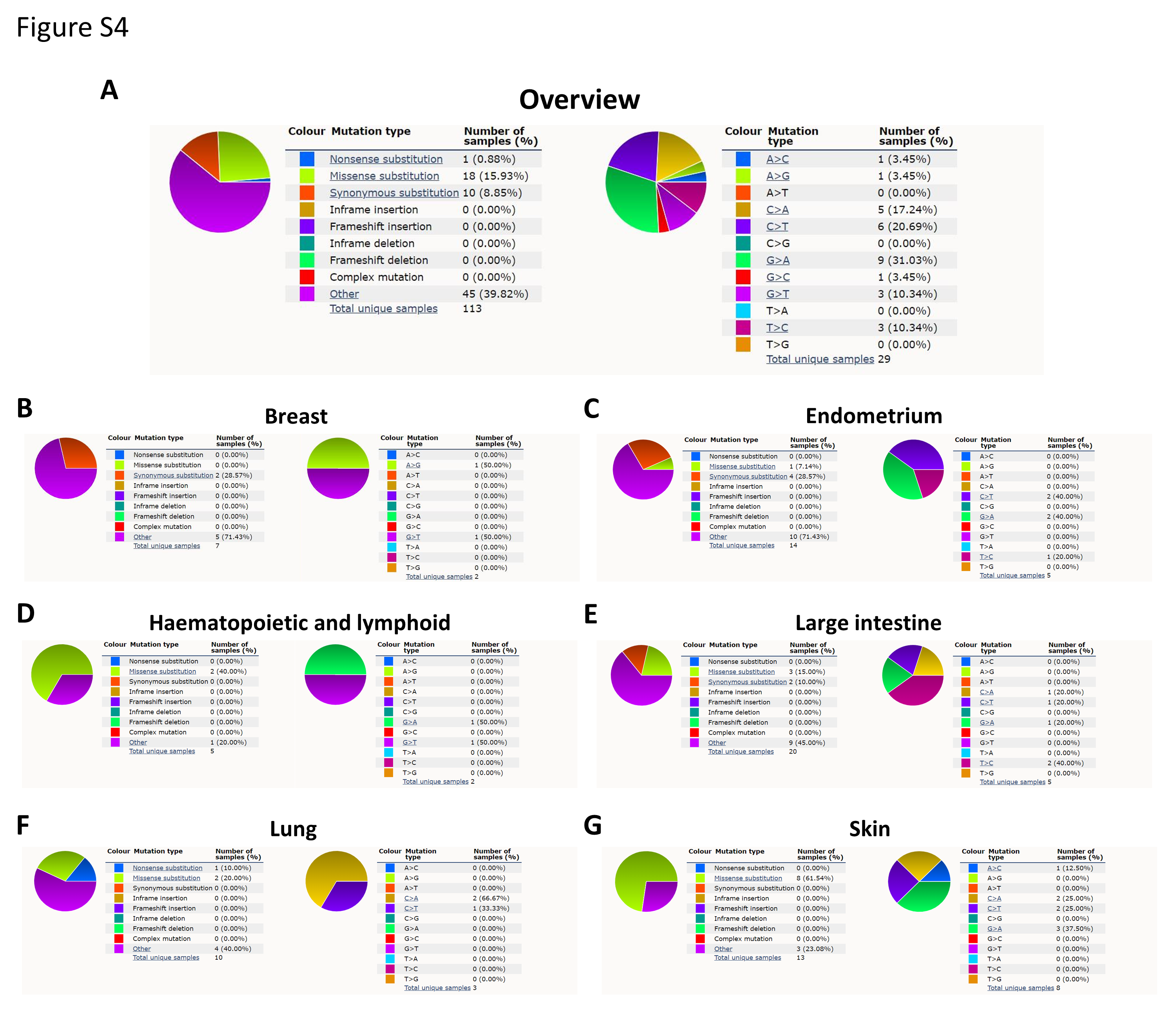
**

**Supplementary Figure S4.** Pie chart of the percentages of *TGFBI* mutation types in human cancers according to the COSMIC database.


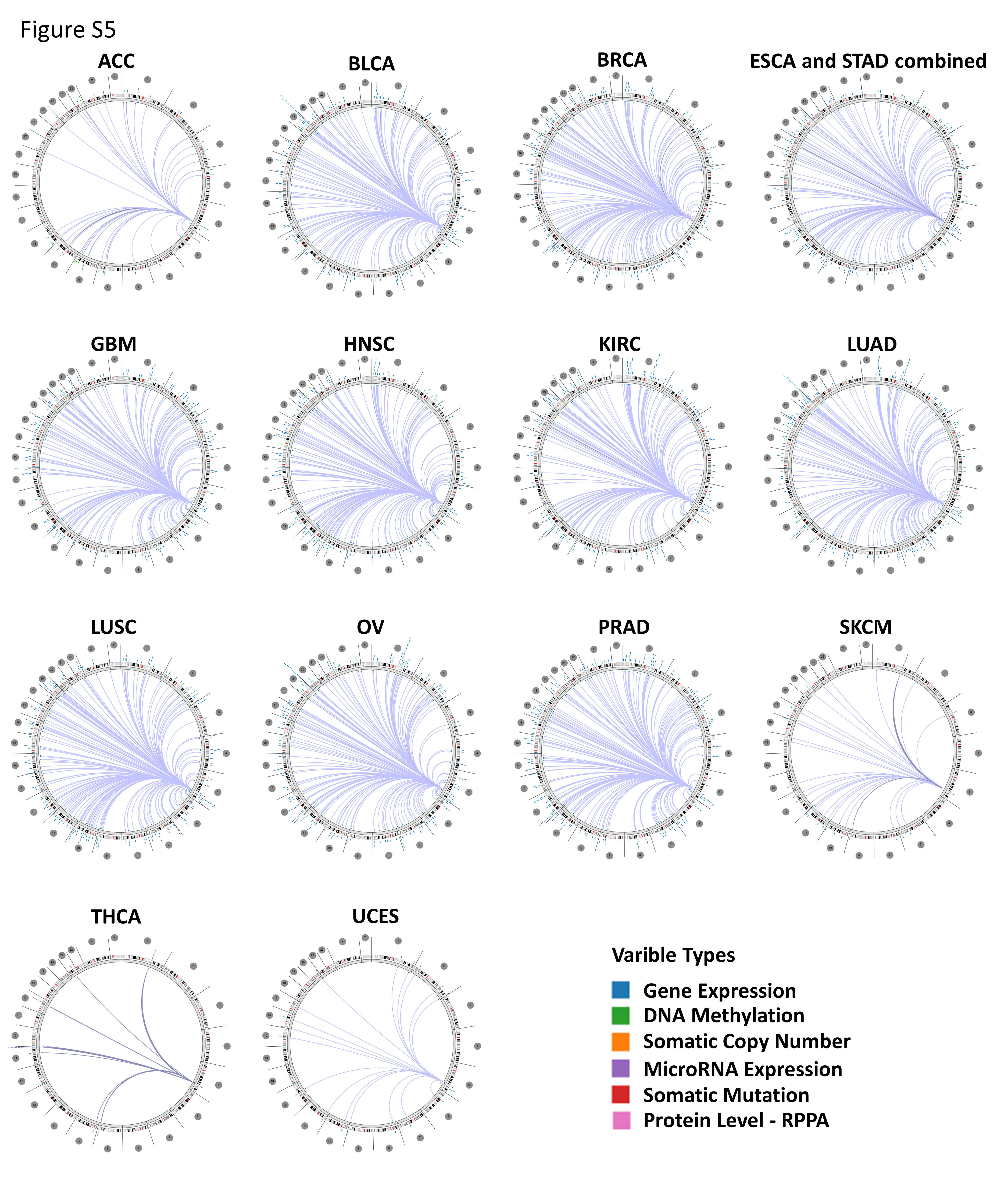


**Supplementary Figure S5.** Genome-wide correlations of *TGFBI* with other signatures using the Cancer Regulome Explorer and TCGA data.

**
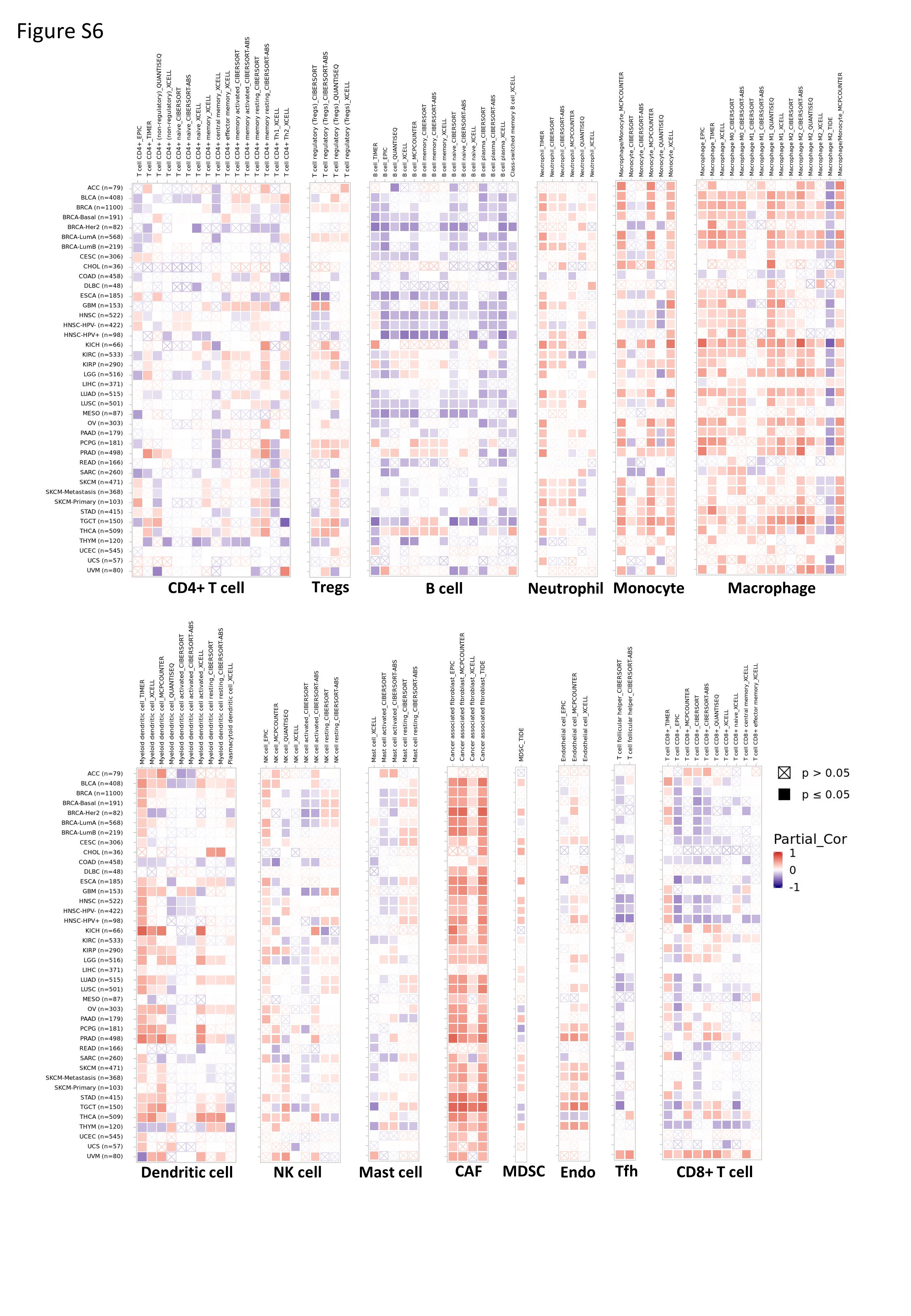
**

**Supplementary Figure S6.** Correlations of *TGFBI* expression with immune cell infiltration in cancers.
